# Sex Differences in Anxiety: An Investigation of the Moderating Role of Sex in Performance Monitoring and Attentional Bias to Threat in High Trait Anxious Individuals

**DOI:** 10.3389/fnhum.2021.627589

**Published:** 2021-05-20

**Authors:** Natalie Strand, Lin Fang, Joshua M. Carlson

**Affiliations:** Department of Psychological Science, Northern Michigan University, Marquette, MI, United States

**Keywords:** attention bias for threat, sex differences, trait anxiety, error-monitoring, event-related potentials, error-related negativity

## Abstract

Anxiety disorders are more predominant in women than men, however there is a lack of understanding as to what neurocognitive mechanisms drive this sex difference. Recent investigation has found a potential moderating role of sex in the relationship between anxiety and the error related negativity (ERN)—a component of error-monitoring that is prevalent in high anxiety individuals—such that females display a positive relationship between anxiety/worry and ERN amplitude. We strove to further explore the influence of sex on the relationship between trait anxiety and performance monitoring, specifically with ERN, as well as extend this work to include another hallmark of anxiety, attentional bias to threat. To meet this end, participants performed the flanker and dot-probe tasks, respectively. We did not find a significant difference in the relationship between attention bias scores and anxiety for female vs. males participants. Furthermore, ΔERN amplitudes were greater in males compared to females, and males had more positive CRN amplitudes than females. There were no significant associations between ERN or ΔERN with anxiety in both male and female participants. However, there was a significant relationship between CRN amplitudes and trait anxiety in male but not female participants. Given these results, the effect of sex on the relationship between components of performance monitoring—namely the CRN and ERN—and anxiety may be more nuanced than the current understanding. Our study was limited to detecting medium to large sized moderation effects. Our findings may be important for future meta-analysis on sex differences in anxiety.

## Introduction

Anxiety disorders are among the most prevalent mental disorders in the advancing world (Demyttenaere et al., 2004). Determining a scientific framework for understanding anxiety disorders and their accompanying symptomatology has become more focused on specific measurable facets or neurocognitive markers of anxiety, including a hyper-vigilance toward threat (Stein and Nesse, 2011), and instability in performance monitoring (Moser et al., 2013). Recently, there has been increased attention paid to the role of sex in moderating neurocognitive markers of different anxiety-related disorders (Larson et al., 2011; Moran et al., 2012; Moser et al., 2016). Females have been found to experience a higher prevalence of both affective and anxiety disorders than males (Kessler et al., 2012). Therefore, it is imperative that studies investigating different facets of anxiety and its neural underpinnings include sex as a variable.

One neurocognitive marker linked to anxiety is the tendency to exhibit a hyperactive neural response to errors (Hajcak et al., 2003; Hajcak and Foti, 2008; Carrasco et al., 2013; Cavanagh et al., 2017). Errors function as a source of threat, leading to greater distress, and subsequent defense mobilization (Hajcak and Foti, 2008; Hajcak, 2012; Proudfit et al., 2013). Anxious individuals tend to worry about threat to current goals, such as achieving a sufficient level of performance, and work to adopt strategies to reduce the anxiety to reach such goals (Eysenck et al., 2007). Evaluating and correcting one's behavior throughout a task is broadly referred to as performance monitoring (Taylor et al., 2007). Error-monitoring involves recognition and correction of errors. The Error-Related Negativity (ERN) is an event-related potential (ERP) with a negative deflection peaking ~100 ms following an incorrect response (Gehring et al., 1993). It originates in the dorsal anterior cingulate cortex (ACC), and appears to involve a distributed network of regions including the prefrontal cortex (PFC) and supplementary motor area collectively involved in error-monitoring (SMA; Taylor et al., 2007; Gehring et al., 2012). Variability in ERN amplitude is linked to greater ACC—SMA functional connectivity (Gilbertson et al., 2021). According to the conflict monitoring theory (Carter et al., 1998; Botvinick et al., 1999, 2001), the ACC detects conflict during response selection, and relays this information to other brain regions involved in cognitive control.

Cognitive deficits in anxious individuals, particularly with processing efficiency, are proposed to impact performance (Eysenck et al., 2007). Furthermore, anxious apprehension (i.e., worry) is thought to moderate cognitive abnormalities in anxiety (Eysenck et al., 2007; Moser et al., 2013). Enhanced ERN in anxious individuals with high worry may indicate higher compensatory effort needed to maintain a standard level of performance (Moser et al., 2013). Thus, the distracting impact of worry leads to inefficient cognitive processing given that anxious individuals utilize greater error-monitoring resources to reach the same level of performance as those without anxiety. In general, the ERN is a consistent and reliable neural marker of anxiety symptomatology (Weinberg et al., 2010, 2012).

The correct-response negativity (CRN) is another performance monitoring potential that occurs around the same time as the ERN, and is thought to reflect a similar process as the ERN, however on correct trials (Vidal et al., 2000, 2003). Similar to the ERN, an enhanced CRN has been found in anxious individuals (Hajcak and Simons, 2002; Hajcak et al., 2003; Endrass et al., 2008, 2010; Moran et al., 2012), therefore it has been proposed that anxiety is related to a general increase in performance monitoring processes originating in the ACC.

Recent investigations into the ERN and CRN have found sex differences. Males exhibit increased ERN amplitudes relative to females (Larson et al., 2011; Fischer et al., 2016; Imburgio et al., 2020). Worry in particular is associated with enhanced ERN and CRN amplitudes in female but not male undergraduates (Moran et al., 2012). Furthermore, meta-analysis indicates that the relationship between anxiety and ERN amplitude is greater in females than males (Moser et al., 2016). Given these results, the ERN and CRN could potentially serve as biomarkers of anxiety in females. Aside from these findings, there is little research examining the moderating role of sex in the relationship between performance monitoring and anxiety.

Another neurocognitive marker of anxiety is an attention bias toward threat-related stimuli (Dalgleish and Watts, 1990; Bar-Haim et al., 2007), which is thought to play a causal role in developing and sustaining anxiety symptoms (MacLeod et al., 1986, 2002). Eysenck et al. (2007) attentional control theory predicts that adverse effects of anxiety on performance occur more often when stimuli are threat-related vs. neutral, such that inhibitory functions are less efficient in anxious individuals in the face of threat-related distractors. At the neural level, the amygdala plays a role in the processing of threat-related information and behavior (LeDoux, 2000; Davis and Whalen, 2001; Rosen, 2004; Davis, 2006; Myers and Davis, 2007), including attentional bias to threat (Anderson and Phelps, 2001; Monk et al., 2004, 2008; Van den Heuvel et al., 2005; Carlson et al., 2009). Furthermore, elevated ACC gray matter volume (Carlson et al., 2012), and amygdala–ACC connectivity (Carlson et al., 2013) are linked to heightened attention bias to threat. Thus, abnormal performance monitoring and attentional bias to threat are both anxiety-related symptoms that appear to share a common neural substrate in the ACC.

Similar to performance monitoring, previous research has also explored sex differences in attention bias. Meta-analysis indicates no sex differences in attentional bias to threat^1^. Thus, sex does not appear to be related to differences in attentional bias. However, to the best of our knowledge, there is no research assessing the effect of sex as a moderator of the relationship between anxiety and attention bias in individuals with high levels of trait anxiety.

The objective of this study was to further investigate sex differences in performance monitoring and attention bias as well as the potential moderating role of sex in the relationship between anxiety and performance monitoring as well as anxiety and attention bias. Thus, we investigated the effect of sex on the relationship between both ERN and CRN and anxiety as well as the relationship between attention bias and anxiety. We hypothesized that male participants would demonstrate a larger ERN amplitude in comparison to female participants, and anticipated a greater association between ERN/CRN and trait anxiety in females than in males. However, we did not expect to find sex differences in attention bias or its relationship with anxiety.

## Methods

### Participants

A sample of 114 individuals were recruited from the university and surrounding community. These participants were involved in a larger clinical trial (NCT03092609) assessing the effect of attention bias modification over a 6-week period, and were subject to the following inclusion criteria: (1) right-handed, (2) 18–42 years of age, (3) normal (or corrected to normal) vision, (4) no current psychological treatment, (5) no recent history of head injury or loss of consciousness, (6) no current psychoactive medications, (7) not claustrophobic, (8) not pregnant, (9) no metal in the body or other MRI contraindications (10) trait anxiety scores ≥40 on the STAI-T, and (11) attentional bias scores ≥7 ms in the dot-probe task.

Participants were excluded from data analysis for having fewer than 8 valid ERN error trials (*n* = 4) or flanker accuracy below 75% (*n* = 3). The final sample included one-hundred and seven individuals 18–38 years old (*M* = 21.81, *SD* = 4.75). Sex was collected according to participants' assigned sex at birth (*n*_female_ = 71, *n*_*male*_ = 36). Using G^*^Power (3.1.9.2), we ran a sensitivity analysis to test for interaction effects in ANCOVA with α = 0.05, power = 0.80, *N* = 107, which indicates that our study was powered to detect medium-to-large effect sizes of *f* ≥ 0.27.

### State-Trait Anxiety Inventory

Trait anxiety scores were collected from the Spielberger state-trait anxiety inventory (STAI) which uses self-report measures of participants' anxiety (Spielberger et al., 1970). The STAI measures both transient (i.e., state) and persistent (i.e., trait) levels of anxiety, and demonstrates high validity, reliability, and discriminative validity between both dimensions (Metzger, 1976). There are 40 questions based on a 4-point likert scale, with 20 questions specifically measuring state anxiety and the other 20 measuring trait anxiety.

### Flanker Task

**Figure 1:**
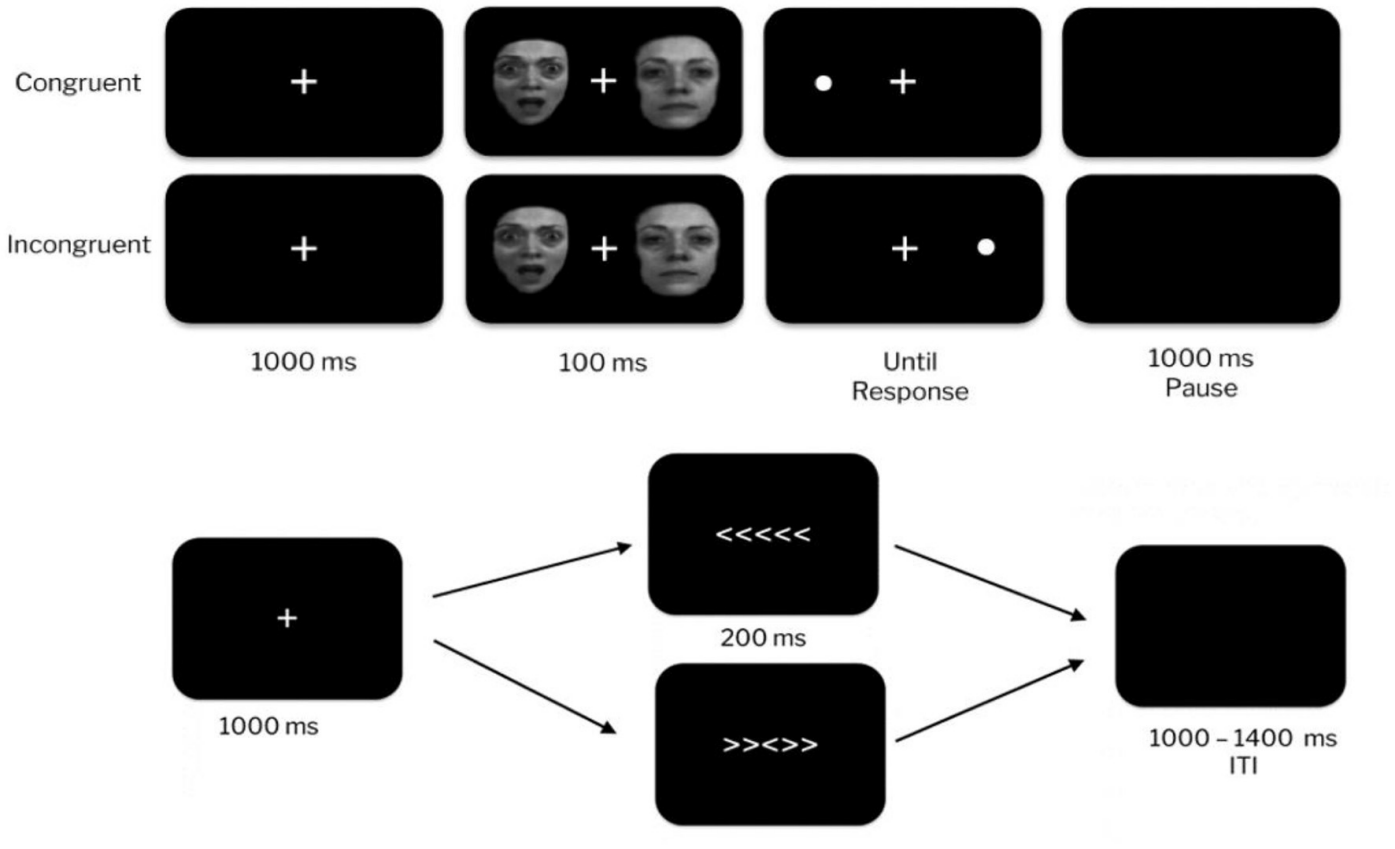
Dot-probe task (top). Examples of congruent and incongruent trials with fearful and neutral face stimuli. Participants respond to the location of the target dot. Attentional bias is measured by faster reaction times on congruent relative to incongruent trials. Arrow flanker task (bottom). Participants were instructed to identify the direction of the center arrow when flanker arrows were compatible (top) or incompatible (bottom).

A modified Eriksen flanker task was administered with E-Prime 3.0 (Figure 1). During each trial, five white, centered, and horizontally positioned arrows were presented for 200 ms after a 1,000 ms fixation cue. Stimuli were presented as either a compatible trial (e.g., < < < < < or > > > > >) or an incompatible trial (e.g., < < > < < or > > < > >), and each trial type had an equal probability of occurring. Arrow stimuli were used because they have been found to exhibit the strongest convergent validity for ERN elicitation (Riesel et al., 2013). Following stimulus presentation, there was a 1,000–1,400 ms inter-trial interval during which participants indicated the direction the center arrow (right or left). The task contained a practice block of 20 trials, and seven subsequent blocks of 60 trials (15 trials of each stimulus type).

Throughout the task, verbal emphasis was placed on both speed and accuracy in order to sufficiently elicit a valid ERN. To meet this standard, participants had to maintain an accuracy level between 75 and 90% for each block (Olvet and Hajcak, 2009; Larson et al., 2010; Pontifex et al., 2010). After each block, the screen displayed the participant's accuracy for that block, and based on their performance, the experimenter provided one of three types of feedback: participants instructed to respond faster in order to commit more errors (accuracy above 90%), they are instructed to respond slower (accuracy below 75%), or they are told that they responded appropriately with balanced speed and accuracy.

### Dot-Probe Task

The dot-probe task was performed with stimuli presented using E-Prime 2.0, and responses recorded *via* the Chronos response box (Psychology Software Tools, Sharpsburg, PA). The task involved presentation of 10 facial stimuli pairs featuring both neutral and fearful facial expressions with 50% female stimuli. Faces were edited to remove hair and presented in gray scale. Of the 20 stimuli, 12 were retrieved from the Karolinska Directed Emotional Face database (Lundqvist et al., 1998), and eight were retrieved from the 3D Facial Emotional Stimuli database (Gur et al., 2002).

As displayed in Figure 1, each trial began with a fixation cue presented for 1,000 ms, and immediately followed by one of three stimulus pairings presented horizontally to the fixation cue for 100 ms. These stimulus pairings were then replaced by a dot, and participants were required to respond to which side of the screen the dot was located on, followed by an inter-trial interval of 1,000 ms. Throughout the task, the participant was seated 59 cm from the monitor and instructed to maintain fixation on the fixation cue.

The dot-probe paradigm consisted of five blocks of 90 trials (450 total trials), with each block containing three equally presented trial types. Such trial types included incongruent trials (dot always appeared behind neutral stimulus in a neutral-fearful stimulus pairing), congruent trials (dot appeared behind fearful stimulus in a neutral-fearful pairing), and neutral-same trials (dot appeared behind neutral stimulus in a neutral-neutral stimulus pairing). EEG data were not collected during the dot-probe task.

### Data Collection, Recording, and Analysis

#### EEG Data

Continuous EEG was recorded during the flanker task using a 64 channel Geodesic Sensor Net (Electrical Geodesics Inc., Eugene OR) with AgCl electrodes placed according to the international 10–20 system. The EEG was recorded *via* Net Station 4.5 software (Electrical Geodesics Inc., Eugene, OR) and was digitized at a sampling rate of 500 Hz. Similar to previous research using the EGI system, electrode impedance levels were kept below 75 kΩ (Rizer et al., 2018; Tunison et al., 2019).

#### Behavioral Data

The dot-probe behavioral data was combined and averaged within E-Prime 2.0 software. Trials with an incorrect response and/or trials with a RT <150 or >750 ms were excluded from analysis in order to eliminate premature responses and lapses in attention (Aday and Carlson, 2019). Attention bias scores were calculated by taking the average reaction times (RT) for both incongruent and congruent trials and subtracting the mean incongruent RT from the congruent RT. Higher scores represent more attentional bias toward threat. Behavioral data from the flanker and dot-probe tasks were combined and averaged within E-Prime 3.0 software.

### EEG Processing and ERP Data Analysis

EEG preprocessing was completed with EEGLAB toolbox v2019.0 (Delorme and Makeig, 2004) and ERPLAB toolbox V7.0.0 (Lopez-Calderon and Luck, 2014). Each continuous EEG file was re-referenced to a mastoid average and bandpass filtered (30 Hz lowpass, 0.1 Hz highpass). Data was then segmented from −500 to 500 ms around the participant's response within the flanker task and subjected to a −400 to −200 ms baseline correction. Segments were separated by participant response (correct or incorrect). An independent component analysis (ICA) was performed on segmented EEG data. ICA was used to isolate artifactual EEG components including eye, muscle, and heart activity, line or channel noise, and other unidentifiable sources from brain activity. By extracting these components from the dataset, one can remove evidence of artifacts without removing other activity of interest (Delorme and Makeig, 2004). After visual inspection of the scalp distributions and activity power spectrum of components for each participant, the components with obvious eyeblink and muscle artifacts were removed. In further artifact rejection, a step function was used to detect voltage exceeding 100 μV within the time period between 500 ms before and 200 ms after the response. Finally, segments were averaged.

Segment counts were inspected to ensure that there were at least eight valid incorrect response segments (Olvet and Hajcak, 2009; Pontifex et al., 2010). Failure to meet this segment count resulted in exclusion from analysis. Segment averages were combined into grand average files, which were visually inspected in order to determine the frontocentral electrode that best represented ERN. Consistent with previous research (e.g., Olvet and Hajcak, 2009; Pontifex et al., 2010), the mean amplitude of the frontocentral electrode, FCz, was extracted 0–100 ms post-response for error (i.e., ERN) and correct trials (i.e., correct related negativity; CRN) for each participant. ΔERN amplitudes were calculated as the ERN–CRN difference.

## Results

### Sex Differences in the Flanker Task

**Figure 2:**
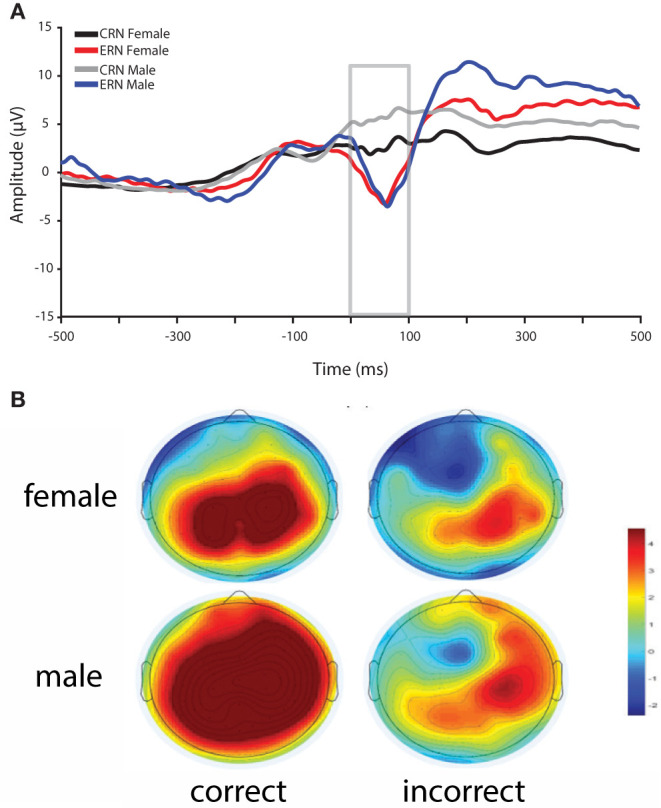
**(A)** ERN and CRN waveforms for incorrect and correct responses for female and male participants. ERN amplitudes did not differ between female and male participants, whereas CRN amplitudes and ΔERN (incorrect–correct) amplitudes were greater in male participants. **(B)** Scalp topography for correct and incorrect response between 0 and 100 ms post-response for females (top) and males (bottom).

Overall, reaction times were quicker for males compared to females, whereas accuracy was greater for females compared to males in the flanker task (see Table 1). In addition, ΔERN amplitudes were greater and CRN were more positive in males compared to females (Figure 2).

**Table 1:** Mean differences (SDs) between females and males on study variables.

**Factor**	**Female (*n* = 71)**	**Male (*n* = 36)**	**Statistics**
Age	21.39 (4.75)	22.64 (4.67)	*t*_(105)_ = 1.29, *p* = 0.20
STAI-trait anxiety	51.37 (7.56)	51.94 (6.68)	*t*_(105)_ = 0.39, *p* = 0.70
STAI-trait worry items	12.30 (3.04)	13.00 (2.80)	*t*_(105)_ = 1.16, *p* = 0.25
STAI-state anxiety	45.34 (10.08)	43.86 (11.35)	*t*_(105)_ = −0.69, *p* = 0.49
Attention bias score (ms)	13.69 (6.69)	16.49 (8.86)	*t*_(105)_ = 1.83, *p* = 0.07
Dot-probe accuracy (%)	98.27 (2.02)	97 (2.66)	*t*_(105)_ = −2.75, *p* = 0.007
Flanker RT (ms)	347.57 (34.18)	330.14 (32.36)	*F*_(1, 105)_ = 6.43, *p* = 0.01
Flanker accuracy (%)	88.91 (4.12)	87.32 (3.41)	*t*_(105)_ = −1.99, *p* = 0.049
ERN amplitude (μV)	−0.91 (4.29)	−0.07 (5.50)	*t*_(105)_ = 0.86, *p* = 0.39
CRN amplitude (μV)	2.92 (3.95)	5.99 (4.50)	*t*_(105)_ = 3.62, *p* <0.001^*^
ΔERN amplitude (μV)	−3.83 (4.37)	−6.06 (4.54)	*t*_(105)_ = −2.47, *p* = 0.015

*STAI, State Trait Anxiety Inventory; ERN, error-related negativity; CRN, correct-related negativity; ΔERN, difference score between ERN and CRN. With the Bonferroni correction applied with 11 comparisons α = 0.0045*.

* *p <0.0045*.

### Sex Differences in the Dot-Probe Task

As can be seen in Table 1, male participants tended to have greater attention bias scores (incongruent—congruent RTs) compared to female participants in the dot-probe task, but this effect was not statistically significant. Females had greater accuracy in the dot-probe task compared to males (see Table 1).

### Sex Differences in the Association With Anxiety

The general linear model was used to assess the interaction between anxiety and sex to determine the moderating role of sex with each of the dependent variables (see Table 2). Sex moderated the association between trait anxiety and CRN amplitudes. There was a positive correlation between CRN amplitudes and trait anxiety in male, but not female participants. Sex did not moderate the associations between trait anxiety and ΔERN or ERN amplitudes. Furthermore, sex did not moderate the association between trait anxiety and any other study variable. The same general pattern was found when only worry-related items from the STAI-T were used (see Table 3; Verkuil and Burger, 2019).

**Table 2:** Test of sex differences in the correlations between trait anxiety and study variables.

**Factor**	**Female STAI-T**	**Male STAI-T**	**Anxiety × sex interaction**
Dot-probe attention bias	−0.12	0.08	*F*_(1, 103)_ = 0.88, *p* = 0.35, $\eta_{\text{partial}}^{2}$ = 0.008
Dot-probe accuracy	−0.07	0.13	*F*_(1, 103)_ = 1.08, *p* = 0.30, $\eta_{\text{partial}}^{2}$ = 0.01
Flanker RT	0.07	−0.09	*F*_(1, 103)_ = 0.59, *p* = 0.44, $\eta_{\text{partial}}^{2}$ = 0.006
Flanker accuracy	−0.25^*^	0.03	*F*_(1, 103)_ = 1.74, *p* = 0.19, $\eta_{\text{partial}}^{2}$ = 0.02
CRN amplitude	0.01	0.37^*^	*F*_(1, 103)_ = 4.01, *p* = 0.048, $\eta_{\text{partial}}^{2}$ = 0.04
ERN amplitude	0.18	0.25	*F*_(1, 103)_ = 0.52, *p* = 0.47, $\eta_{\text{partial}}^{2}$ = 0.005
ΔERN amplitude	0.17	−0.07	*F*_(1, 103)_ = 1.18, *p* = 0.28, $\eta_{\text{partial}}^{2}$ = 0.01

*STAI, State Trait Anxiety Inventory; ERN, error-related negativity; CRN, correct-related negativity; ΔERN, difference score between ERN and CRN*.

* *Indicates bivariate pearson correlation p <0.05*.

**Table 3:** Test of sex differences in the correlations between worry only items and study variables.

**Factor**	**Female STAI-T worry**	**Male STAI-T worry**	**Anxiety × sex interaction**
Dot-probe attention bias	−0.15	0.09	*F*_(1, 103)_ = 1.33, *p* = 0.25, $\eta_{\text{partial}}^{2}$ = 0.01
Dot-probe accuracy	−0.09	0.004	*F*_(1, 103)_ = 0.14, *p* = 0.71, $\eta_{\text{partial}}^{2}$ = 0.001
Flanker RT	−0.03	0.02	*F*_(1, 103)_ = 0.47, *p* = 0.83, $\eta_{\text{partial}}^{2}$ = 0.000
Flanker accuracy	−0.14	0.11	*F*_(1, 103)_ = 1.32, *p* = 0.25, $\eta_{\text{partial}}^{2}$ = 0.01
CRN amplitude	−0.10	0.45^*^	*F*_(1, 103)_ = 8.66, *p* = 0.004, $\eta_{\text{partial}}^{2}$ = 0.08
ERN amplitude	0.16	0.37^*^	*F*_(1, 103)_ = 2.31, *p* = 0.13, $\eta_{\text{partial}}^{2}$ = 0.02
ΔERN amplitude	0.24^*^	0.003	*F*_(1, 103)_ = 1.20, *p* = 0.28, $\eta_{\text{partial}}^{2}$ = 0.01

*STAI, State Trait Anxiety Inventory; ERN, error-related negativity; CRN, correct-related negativity; ΔERN, difference score between ERN and CRN*.

* *Indicates bivariate pearson correlation p <0.05*.

## Discussion

The purpose of this study was to further investigate sex as a moderator in the relationship between symptoms of anxiety and performance monitoring in addition to anxiety and attention bias. Our findings may contribute to future meta-analyses on sex differences in anxiety and highlight the significance of assessing sex differences in future research.

### Performance Monitoring

Consistent with previous research, ΔERN amplitudes were greater in males compared to females (Larson et al., 2011; Fischer et al., 2016; Imburgio et al., 2020). In particular, in our data, this difference is driven by more positive CRN amplitudes in males relative to females with no difference in ERN. This general pattern is consistent with a recent study that found gender effects on the CRN and ΔERN, but not ERN (Imburgio et al., 2020). However, earlier studies found that sex (Larson et al., 2011) or gender (Fischer et al., 2016) differences were more related to the ERN. The reason for these discrepancies is unclear. Yet, despite these inconsistencies, it is clear that ΔERN amplitudes are greater in males relative to females. In fMRI research, increased ACC activity has been found in males compared to females during a stop-signal task, where comparable behavioral performance was demonstrated for both sexes (Li et al., 2006). Thus, elevated ACC activity in males may underlie enhanced ΔERN amplitudes. Given that increased ΔERN amplitudes in males has been consistently observed, future research should explore the underlying causes of this effect (e.g., ACC activity), and continue to investigate the potential impact of anxiety.

Furthermore, we found a positive correlation between CRN amplitudes and trait anxiety in male, but not female participants. This is inconsistent with previous research that found a relationship in females, but not males (Moran et al., 2012). However, this earlier study specifically investigated worry, which has been found to be related to the CRN and ERN in females (Moran et al., 2012; Moser et al., 2016). In contrast, a meta-analysis examining CRN and anxiety found no significant relationship (Moser et al., 2013). The correlation between CRN and anxiety in males could potentially be attributed to performance monitoring on correct response trials and motivation to perform more accurately, especially for individuals with higher anxiety. To the best of our knowledge, this is the first evidence suggesting that trait anxiety in males is linked to CRN amplitude. Given the unexpected nature of this association, this result should be interpreted with caution and replication is warranted.

There were no significant associations between ERN or ΔERN with anxiety in either males or females. This is an unexpected result, given that previous research suggests the relationship between anxiety and ERN amplitudes is greater in females than males (Moser et al., 2016). A lack of significant association in females could be due to several underlying factors.

First, although initial evidence points to sex differences in the relationship between anxiety and the ERN (Moran et al., 2012; Moser et al., 2016), there is a growing understanding that this association is more nuanced, and further replication is necessary to support these claims. In recent attempts to replicate sex differences in the relationship between the ERN and anxiety, there are opposing results, suggesting a lack of clarity in the association between these variables. A study assessing the relationship between anxiety dimensions and ERN in a non-clinical sample found that gender did not moderate the association between anxious apprehension and ERN (Härpfer et al., 2020). In addition, a recent multi-site study was unsuccessful in replicating the work of Moser et al. (2016) across sites, including one study that found effects in the opposite direction (Moser et al., 2019). Thus, it is becoming clear that the differential association between ERN and anxiety across sexes is not universally observed. Here, we demonstrate that in highly anxious individuals with high attentional bias, no relationship appears to exist or perhaps there are sex differences related to the CRN in males.

Second, it is unclear which specific factors mediate the ERN-anxiety relationship in both sexes. A study by Tanovic et al. (2017) found that in contrast to worry, rumination was associated with an *attenuated* ERN. They suggest that individuals who ruminate respond to aversive events by enhanced engagement in the negative thoughts that are brought upon by an aversive stimulus, thus leading to a diminished ERN amplitude. This study did not consider sex differences, however given the higher prevalence of rumination in females (Johnson and Whisman, 2013), it is surprising that an attenuated ERN has not carried over into other studies that found significance in the ERN-anxiety relationship for females. One possible explanation is that anxious apprehension includes both elements of worry and rumination. These two variables may affect distinct cognitive systems, and future research should investigate the moderating role of sex in ERN-worry and ERN-rumination relationships separately to help gain a more clear understanding of the specific components that affect the broad ERN-anxiety relationship in females vs. males.

Third, inconsistency in the operationalization of anxiety, namely our usage of the STAI questionnaire may have contributed to the inability to replicate the ERN-anxiety relationship in females. Prior investigations into sex differences in this relationship typically do not rely on the STAI. Moran et al. (2012) found the relationship between worry and ERN to be specific to females, using the Penn State Worry Questionnaire (PSWQ; Meyer et al., 1990). In addition, Moser et al. (2013) found an association between ERN and anxious apprehension, but their methodology constituted several questionnaires that specifically distinguished “anxiety” as the primary concept measured. In comparison to these studies, our inclusion of the STAI may have yielded different results due to its incorporation of worry components in fewer questions, and a secondary focus on apprehension. When specifically examining worry-related items in the STAI-T, sex did not moderate the association between anxiety and performance monitoring (Table 3).

Fourth, behavioral results from the flanker task indicate that reaction times were quicker for males compared to females, while accuracy was greater for females compared to males (see Table 1). In addition, there was a negative correlation between anxiety and flanker accuracy in females, but not males. This moderation effect did not reach statistical significance (see Table 2). Yet, these differences in behavior may impact sex differences in ERPs. It is possible that males and females took a different approach to the task, thus impacting the relationship between anxiety and ERN/CRN across sexes. Note that earlier work showing sex differences in the relationship between the ERN and anxiety (e.g., Moran et al., 2012) did not observe behavioral differences, which makes direct comparisons between these studies difficult.

### Attentional Bias

The presence of attention bias in anxious individuals is a robust phenomenon (Dalgleish and Watts, 1990). However, sex differences in attention bias and its relationship with anxiety has been overlooked. Consistent with the findings of a recent meta-analysis (Campbell and Muncer, 2017), we found no evidence for sex differences in attentional bias to threat in individuals with high trait anxiety. Thus, attentional bias may not be a suitable measure for investigating sex differences in anxiety, however further research is necessary to elucidate this relationship.

### Limitations and Conclusions

Our study was only powered to detect medium to large sized moderation effects. Therefore, our null results are not definitive and should be interpreted within the context of the larger literature. Our findings may contribute to future meta-analyses and/or synthesis of the literature in this area. Moreover, sex differences may appear throughout the lifespan due to changes in hormone levels during pregnancy, menopause, as well as an individual's menstrual cycle (Gordon and Girdler, 2014), which may influence ERN amplitudes (Mulligan et al., 2019). The use of hormonal birth control can also affect the extent to which sex plays a moderating role (Petersen et al., 2014). Our study included individuals with heightened levels of anxiety and attention bias, which limits the ability to generalize our findings to low anxiety individuals with low levels of attention bias. Our sample also consisted of more female than male participants, and was primarily young adults, which further limits the generalizability of these findings.

Our study investigated sex differences in performance monitoring and attention bias as well as the moderating role of sex in their relationship with anxiety. In line with our predictions, males exhibited a larger ΔERN in comparison to females. Furthermore, as expected, there were no significant sex differences in attention bias and sex had no effect on the relationship between anxiety and attention bias. In contrast to previous research (Moser et al., 2016), we did not find an association between ΔERN and anxiety in females. Unexpectedly, we found a relationship between CRN amplitude and anxiety in males. Further research is warranted to elucidate sex differences in the relationship between anxiety and performance monitoring.

## Data Availability Statement

The raw data supporting the conclusions of this article will be made available by the authors, without undue reservation.

## Ethics Statement

The studies involving human participants were reviewed and approved by Northern Michigan University Institutional Review Board (IRB). The patients/participants provided their written informed consent to participate in this study.

## Author Contributions

NS drafted the manuscript. NS and LF assisted in data collection. LF processed the data. LF and JC analyzed the data and provided critical feedback during manuscript preparation. All authors contributed to the article and approved the submitted version.

## Conflict of Interest

The authors declare that the research was conducted in the absence of any commercial or financial relationships that could be construed as a potential conflict of interest.

## Footnotes

^1^Note, however, that a recent study by Carlson et al. (2019) found a relationship between sex and *temporal dynamics in attention bias* (but not static/global measures of attention bias). Females exhibited enhanced biases towards and away from emotional stimuli, as well as higher variability in attention bias to both positive and negative emotional stimuli (but see Carlson and Fang, 2020 for null sex effects).
